# Prevalent Seasoning and Cooking Fats, Arterial Stiffness and Blood Lipid Pattern in a Rural Population Sample: Data from the Brisighella Heart Study

**DOI:** 10.3390/nu12103063

**Published:** 2020-10-07

**Authors:** Arrigo F.G. Cicero, Federica Fogacci, Elisa Grandi, Elisabetta Rizzoli, Marilisa Bove, Sergio D’Addato, Claudio Borghi

**Affiliations:** 1 Hypertension and Atherosclerosis Research Group, Medical and Surgical Sciences Department, University of Bologna, 40138 Bologna, Italy; federicafogacci@gmail.com (F.F.); elisa.grandi@unibo.it (E.G.); elisabetta.rizzoli@unibo.it (E.R.); marilisa.bove@aosp.bo.it (M.B.); sergio.daddato@unibo.it (S.D.); claudio.borghi@unibo.it (C.B.); 2 IRCCS Policlinico di S. Orsola, 40138 Bologna, Italy

**Keywords:** cooking fats, epidemiology, blood pressure, arterial stiffness, metabolic parameters

## Abstract

*Background*: Dietary fats have been variably associated with the risk of cardiovascular disease. The aim of our study was to evaluate the association between everyday mainly used dietary fats in cooking and as seasoning and hemodynamic and lipid parameters. *Methods*: For this study, we selected from the Brisighella Heart Study cohort subjects who were not treated with antihypertensive drugs and report with certainty their daily mean intake of dietary fats in cooking and as seasoning. Depending on the main source of dietary fat, the involved subjects were classified as prevalent extra-virgin olive oil (EVO) users, prevalent corn oil users, prevalent users of different vegetable oils and prevalent animal fat users, and we compared their characteristics. *Results*: Everyday consumption of EVO as a main seasoning and cooking fat source was significantly associated to lower body mass index, visceral adiposity index, blood pressure, arterial stiffness, and cholesterolemia, when compared with predominantly animal fat users. Corn oil users also had lower blood pressure, arterial stiffness, and cholesterolemia, when compared with predominantly animal fat users, as well. In particular, in an age and systolic blood pressure adjusted model, the predictors of carotid-femoral pulse wave velocity were the prevalent use of EVO (RR = 0.84, 95% CI 0.67–0.94 vs. other prevalent fat sources), LDL-Cholesterol (RR = 1.12, 95% CI 1.02–1.42), serum uric acid (RR = 1.21, 95% CI 1.09–1.54) and estimated GFR (RR = 0.77, 95% CI 0.59–0.99). *Conclusions*: According to our findings, the choice of everyday seasoning and cooking fat is associated with a different metabolic and haemodynamic pattern.

## 1. Introduction

In accordance with the most recent evidence, a healthy diet is the cornerstone of cardiovascular disease prevention [1]. A calorie-balanced Mediterranean diet has been considered one of the most desirable dietary patterns, being associated with a relatively low risk of developing cardiovascular diseases and other chronic degenerative affections [2,3,4]. Furthermore, adherence to a Mediterranean diet has been related to a trend towards a lower risk of all-cause mortality [5].

The Mediterranean diet is characterized by a large intake of plant-based foods (including fruits, vegetables, legumes and nuts) and extra-virgin olive oil (EVO), widely used both as a seasoning and food ingredient, with a generally high monounsaturated/saturated fat (MUFAs/SFAs) ratio. Additional components include a low-to-moderate intake of red wine, a high consumption of whole grains and cereals, a moderate intake of milk and dairy products, a low consumption of meat and its derivates and a large amount of fish intake [6]. However, most of the benefits of the Mediterranean diet on human health have been specifically related to EVO consumption [7]. As a matter of fact, diets rich in olive oil polyphenols seem to be related with reduced biomarkers of oxidative stress and inflammation and exert a beneficial effect on lipid pattern and arterial blood pressure (BP) control [8].

A standard everyday consumption of 25 mL EVO has already been associated with an improvement in the total fatty acid composition of low-density lipoproteins (LDL) and with an increased resistance of LDL-cholesterol (LDL-C) toward oxidative stress [9].

Even though a significant decrease in plasma levels of LDL-C was observed when dietary SFA were replaced by oils rich in both MUFAs and polyunsaturated fatty acids (PUFAs) (i.e., corn, soy, sunflower oils) [10], the comparative effect of different vegetable oils on plasma lipids has not been definitively clarified yet. As a matter of fact, even if some evidence supports a greater LDL-C lowering effect of corn oil than olive oil [11,12], other studies yielded results in the opposite direction [13]. However, few data are available on the long-term effect of seasoning and cooking fats on hemodynamic parameters.

In this context, the aim of our study was to evaluate in a well-characterized sample of population the association between everyday mainly used dietary fats in cooking and as seasoning and hemodynamic and lipid parameters.

## 2. Materials and Methods

The Brisighella Heart Study (BHS) is a cohort active since 1972 on a randomized sample, which is representative of the population of Brisighella, a rural North-Italian village. At the baseline, it involved 2939 Caucasian subjects (1491 men and 1448 women), aged 14–84 and without history of cardiovascular disease at enrolment. The complete study protocol has been previously described elsewhere [14]. Briefly, participants were clinically evaluated at baseline and every 4 years thereafter, by collecting a large setting of clinical data and biochemical parameters. Mortality and morbidity data, as well as the incidence of the main cardiovascular risk factors, were recorded throughout the entire study.

The study has been carried out in agreement with the declaration of Helsinki and the protocol has been approved by the institutional ethical board of the University Hospital of Bologna (Code: BrixFollow-up_1972-2024). All involved subjects signed an informed consent form.

For every subject we recorded a detailed personal and family history (with specific attention to lifestyle and dietary habits, smoking status and pharmacological treatments), a physical examination (including anthropometric data), resting blood pressure and heart rate, a fasting blood sample and a 12-lead electrocardiogram (Minnesota-coded).

Dietary habits were investigated by the use of the Dietary Quality Index, a validated semiquantitative questionnaire investigating the usual food intake of 18 food items grouped in three food categories over the past year [15,16]. Since the Dietary Quality Index does not clearly differentiate between all the considered fats used by the Brisighella citizens, we added to the questionnaire a specific question to better differentiate the main seasoning and cooking fats used in the everyday life, where seasoning fats were defined as not cooked raw fats used on top of salads or meat (oils or creams), and cooked fats the ones added to food during high temperature exposition. The information on diet and life-style were all sampled by trained personnel; no questionnaire was self-administrated.

Waist circumference was measured as the narrowest body diameter between the arcus costarum and the crista iliaca. Height was evaluated with the person standing erect, bare foot together and eyes directed straight ahead. Weight was measured twice, and the average of these two measures was used. Body mass index (BMI) was calculated as weight in kilograms divided by height in meters squared (kg/m^2^). Visceral Adiposity Index was calculated using the formula proposed by Amato et al. [17] Systolic (SBP) and diastolic blood pressure (DBP) were assessed with a standard sphygmomanometer three times at 1-min interval, with the subject in the seated position and after 5 min of quiet rest. The average value of the three measurements was taken as individual blood pressure value.

Biochemical analyses were carried out on venous blood from the basilic vein. Subjects were fasted for at least 12 h at the time of sampling. All available routine laboratory parameters were sampled with standardized methods by trained personnel [18], evaluating fasting plasma glucose (FPG), total cholesterol (TC), triglycerides (TG), high-density lipoprotein cholesterol (HDL-C), LDL-C, apolipoprotein AI (apoAI), apolipoprotein B-100 (apoB), lipoprotein(a), aspartate aminotransferase (ALT), alanine aminotransferase (AST), gamma-glutamyl-transferase (GGT), total bilirubin (TB), creatinine, estimated glomerular filtration rate (eGFR), SUA and creatinine phosphokinase (CPK). Hepatic Steatosis Index (HSI) has been calculated with the formula validated by Lee et al. [19] and already tested on the BHS cohort [20].

Peripheral and central BP, Augmentation Index (AIx) and carotid-femoral pulse wave velocity (cfPWV) were measured noninvasively by the Vicorder^®^ apparatus (Skidmore Medical Ltd., Bristol, UK), which is a validated, commercially available, operator-independent device which determines brachial oscillometric BP using a cuff placed around the upper arm. All the measurements were recorded with the subject in the supine resting position. Brachial pressure waveforms were recorded with the same cuff using a volume displacement technique. Central BP parameters were derived from brachial BP waveforms self-calibrated to brachial SBP and brachial DBP, according to a previously described brachial-to-aortic transfer function [21,22]. The Vicorder^®^ apparatus was already used in other epidemiological studies, as well [23,24].

During the last BHS Survey, we consecutively evaluated 1,652 subjects (male: 46.6%, female: 53.4%). For this analysis, we selected 1203 subjects not using antihypertensive drugs and who reported with certainty their daily mean intake of dietary fats in cooking and as seasoning. We further excluded 119 subjects who used together different types of dietary fats, as seasoning or in cooking without a specific preference.

Depending on the main source of dietary fat (>50% of total seasoning and cooking fats), involved subjects were classified as prevalent EVO users, prevalent corn oil users, prevalent users of other vegetable oils (mixed seeds oils, 62%; sunflower oil, 24%; other oils, 14%), and prevalent animal fats users (cow butter, 54%; pork fat, 46%). The 50% cut-off was chosen being well known that in that geographical area different type of fats are usually used together by habit, as seasoning and during cooking. The relatively high prevalence of corn oil users is due to the involvement of the BHS cohort in a population educational program encouraging substitution of animal fats with corn oil [25], whereas EVO is widely used as it is produced there.

A full descriptive analysis was carried out for all the considered variables. A Kolmogorov-Smirnov normality test was performed for all the continuous variables. All of the continuous variables were compared among the different cooking fat users by ANOVA followed by the Tukey post-hoc test. Non-normally distributed parameters were then log-transformed before going on with the analyses. As an additional test, we carried out a multiple regression analysis to check the predictors of cfPWV. All tests were carried out using SPSS 23.0 for Windows (IBM Corporation, Armonk, NY, USA). A significance level of 0.05 was considered as valid for every test.

## 3. Results

Prevalent EVO users (mean age: 58 ± 6 years old; female: 54%), prevalent corn oil users (mean age: 56 ± 8 years old; female: 56%), prevalent users of other vegetable oils (mean age: 59 ± 7 years old; female: 51%), and prevalent animal fat users (mean age: 60 ± 4 years old; female: 50%) had similar age and gender distribution (*p* > 0.05). Subjects in treatment with lipid-lowering drugs and/or antidiabetic agents were about 4% and 1% of the overall sample and were similarly distributed among the considered subgroups (*p* > 0.05).

Table 1 resumes the main anthropometric characteristics of the subjects with different dietary habits in fat sources. Subjects mainly consuming EVO had significantly lower BMI and Visceral Adiposity Index compared with predominantly animal fat consumers. Animal fat consumers had significantly higher Visceral Adiposity Index compared with predominantly corn oil consumers.

Table 2 resumes the main characteristics of the diet composition of the considered population subgroups. The total energy intake and the distribution of energy from carbohydrates, proteins and fats was similar among the subgroups. Subjects predominantly using animal fats for seasoning and cooking assumed significantly more SFAs a cholesterol. Predominantly EVO consumers assumed more MUFAs and fibers, while the ones consuming more corn oil assumed more PUFAs.

Table 3 summarizes the main haemodynamic characteristics of the subjects with different predominant seasoning and cooking fat habits. Subjects mainly consuming EVO had significantly lower SBP, ABP and AIx compared with consumers of different vegetables oils than EVO and corn oil. EVO consuming subjects had also significantly lower SBP, PP, MAP, ABP, APP and AIx compared with predominantly animal fats users, as well as higher cfPWV. Finally, Animal fats users had also significantly higher PP and AIx, and lower cfPWV compared with predominantly corn oil users. In an age and SBP adjusted model, cfPVW was significantly predicted by the prevalent use of EVO (RR = 0.84, 95% CI 0.67–0.94 vs. other prevalent fat sources), LDL-C (RR = 1.12, 95% CI 1.02–1.42), SUA (RR = 1.21, 95% CI 1.09–1.54) and eGFR (RR = 0.77, 95% CI 0.59–0.99).

Table 4 summarizes the main glyco-lipidic characteristics of the subjects with different predominant fat cooking habits. Subjects mainly consuming EVO had significantly lower FPG, TC, LDL-C, TG, Lp(a) and higher HDL-C and ApoAI levels compared with those consuming animal fats. FPG was lower and HDL-C and ApoAI levels were also higher when compared with the ones predominantly consuming corn oil or other vegetable oils. Subjects predominantly consuming animal fats had also higher TC, LDL-C, ApoB and Lp(a) level when compared to those consuming corn oil or other vegetable oils.

Table 5 summarizes the main liver and renal characteristics of the subjects with different predominant fat cooking habits. Subjects mainly consuming EVO had significantly lower HSI and SUA compared with subjects predominantly consuming animal fats.

## 4. Discussion

Most of the health benefits of the Mediterranean diet have been related to its specific content in unsaturated fatty acids [26]. In our study, which involved a large sample of general population, we observed that subjects everyday mainly using animal fats in cooking and as seasoning had worse anthropometric, metabolic and hemodynamic parameters compared to mainly EVO consumers. Corn oil use was associated with a better profile compared to animal fat users, though not EVO consumers.

The positive impact of EVO on anthropometric parameters has recently been confirmed by a meta-analysis of 11 clinical trials, showing that diets rich in olive oil are keener to favour a mild but significant weight loss compared to other oils [27]. In our cohort, we confirmed this effect on VAI, a validated marker of visceral fat function associated with cardiovascular risk [19].

In the BHS cohort, EVO users in everyday seasoning and cooking habits had a better BP profile than animal fats consumers. This could be at least partly due to the fact that EVO consumption is associated with an improved endothelial function, mainly secondary to a reduction in the systemic inflammation [28]. A similar effect on central BP, a main determinant of cfPWV, has been also observed in a recent randomized clinical trial [29]. In this context, our finding on arterial stiffness is of particular interest, since a 1 m/s increase in cfPWV has been associated with an increased relative risk (RR) of developing cardiovascular events (RR = 1.12, 95% CI: 1.07–1.18) and cardiovascular disease mortality (RR = 1.09, 95% CI: 1.04–1.14) [30]. However, a recent randomized clinical trial did not indicate a short-term effect of EVO on arterial stiffness [31], suggesting that the positive association we observed is likely related to a long-life (or at least long-term) exposure.

In clinical trials, n-3- and n-6-rich plant oils are more effective in reducing LDL-C and total cholesterol (TC) compared with olive oil [32]. However, the positive impact of EVO on lipid profile that we observed in comparison with the other fat sources confirms the results of a recent meta-analysis of 27 randomized clinical trials involving 1089 subjects [33], in particular as regards the effect on plasma HDL-C level. Moreover, in our cohort, EVO use was associated with lower level of HSI, and –consequently- to a lower probability to be affected by non-alcoholic fatty liver disease [19].

Some metabolic and hemodynamic parameters were also better in subjects mainly consuming corn oil, than in those prevalently using animal fats, as expected from literature data [34]. However, the EVO global impact on cardiometabolic parameters was better than all other comparators. Of course, other components of the Mediterranean diet could have contributed to the observed effects, even if the considered subgroups mainly differed for the seasoning and cooking fats than for other nutrients [35]. In particular, the different intake in whole grain pasta rich in bioactive peptides or of red wine rich in resveratrol, or other food rich in flavonoids could influence the metabolic and vascular health [36].

We acknowledge that our investigation has some main limitations. Firstly, the sample size of the considered subgroups of participants was relatively small. However, the selected subsamples were age and sex matched with the global BHS cohort. The identification of subgroups created unbalanced samples, being the mainly consumers of EVO more than the other ones. However, this is representative of the seasoning and cooking fat use by the Brisighella citizens. Moreover, we splitted the group of prevalent corn oil users from the one of other non-EVO vegetable oils users, because the corn oil users were largely represented and because it has been supposed possible health advantage [32]. Finally, grouping the non-EVO vegetable oils the animal fat user groups should have been strongly unbalanced comparing the other two groups. Secondly, the transversal design of the study does not allow us to conclude for a cause-effect relationship. Then, the selection of a subgroup of subjects with specific characteristics reduces the possibility to infer the obtained results to unselected populations. However, we think that excluding from the study all subjects actively treated with antihypertensive drugs reduced the possibility that hemodynamic parameters (and in particular arterial stiffness) were modified from different factors than the ones we were investigating in physiological condition. As a matter of fact, vasodilating agents and diuretics might rapidly reduce central and peripheral blood pressure and the hypertensive patients in the BHS cohort assume different drugs at different dosages and moment of the day. Of course, even lipid-lowering drugs and antidiabetic drugs could have modified arterial stiffness and plasma lipid levels, but their effect on arteries is long-term, not depending by the moment of the day. Moreover, after exclusion of the treated hypertensive subjects, the prevalence of these treatments was relatively low and their use pattern similar among the considered subgroups. Finally, they did not acutely modify the central blood pressure measurement depending on the molecule and the timing of assumption, contrarily to what antihypertensive treatments can do. The administration of the Dietary Quality Index given just at the time of the visit is a further limitation of the analysis, since they give no information about the subjects’ eating habits over time, even if the population of Brisighella has a dietary pattern which is relatively homogeneous and constant [37]. Then, we acknowledge that the added question to better differentiate the main seasoning and cooking fats used in the everyday life was not part of the validated Dietary Quality Index questionnaire. Moreover, the dietary pattern of the single considered subgroups seemed to be relatively similar. However, the questionnaire was validated based on a 12 month recall and in a large cohort was able to predict the development of an unfavorable cardio-metabolic profile [38]. This could be a strength of the study, since the cooking fat sources are less influenced by the season than other dietary choices. Another defect of the questionnaire is that it does not consider the way to use the oil, if fresh or cooked [39]. This has some possible consequences on parameters like SUA, because cooked oil could be less protective against oxidative stress, and usually EVO is less used for cooking than other oils. Furthermore, we analyzed group by prevalent seasoning and cooking fat use, while in real-life the most part of subjects did not use only one kind of fat: this could have underestimated the positive effects of EVO and other vegetable oils on the investigated parameters. Finally, we did not investigate oxidative parameters that could have modified in a more impressive way in the prevalent EVO consumers.

Beyond all these limitations, our observations support the role of everyday choice in term of everyday seasoning and cooking fat in determining the plasma level of glucose and lipid levels, but also of arterial health in term of degree of arterial stiffness. This should reinforce the application of the guidelines suggestion as regards the improvement of dietary habits to prevent cardiovascular diseases.

## 5. Conclusions

In conclusion, according to our findings, the choice of everyday seasoning and cooking fat is associated with a different lipid and haemodynamic pattern. In particular, the prevalent use of EVO as everyday seasoning or cooking fat source is associated with an overall healthier lipid and hemodynamic pattern in a large rural population sample. Corn oil use seems to be a possible healthy alternative.

## Figures and Tables

**Table 1:** Anthropometric characteristics of subjects consuming different predominant cooking fats in everyday meals.

	Extra-Virgin Olive Oil (*N*. 501)	Corn Oil (*N*. 253)	Other Vegetable Oils (*N*. 107)	Animal Fats (*N*. 103)	ANOVA *p*-Value
Body mass index (kg/m^2^)	25.6 ± 2.3 *	26.2 ± 2.4	26.5 ± 2.7	26.8 ± 2.6	0.134
Waist circumference (cm)	89.1 ± 6.2	90.4 ± 9.3	91.3 ± 8.1	91.9 ± 7.5	0.472
Waist to Hip ratio	0.92 ± 0.10	0.90 ± 0.16	0.90 ± 0.14	0.90 ± 0.12	0.583
Visceral Adiposity Index	1.8 ± 1.0 *	1.8 ± 1.1 *	1.9 ± 1.7	2.2 ± 1.4	0.041

* *p* < 0.05 vs. Animal fats (Analysis of Variance—ANOVA—followed by Tukey post-hoc test; all variable were normally distributed).

**Table 2:** Main diet composition characteristics of subjects consuming different predominant cooking fats in everyday meals.

	Extra-Virgin Olive Oil (*N*. 501)	Corn Oil (*N*. 253)	Other Vegetable Oils *(N*. 107)	Animal Fats (*N*. 103)	ANOVA *p*-Value
Total energy intake (kcal)	2075.2 ± 235.8	2056.8 ± 291.3	2175.5 ± 240.1	2216.9 ± 287.9	0.094
% of energy from carbohydrate	52.0 ± 3.5	49.5 ± 4.1	51.3 ± 4.2	49.9 ± 4.3	0.421
% of energy from protein	16.4 ± 2.1	15.3 ± 2.3	15.1 ± 2.2	16.9 ± 2.8	0.641
% of energy from fat	31.2 ± 2.6	34.1 ± 2.1	34.0 ± 2.4	33.6 ± 3.1	0.327
SFAs (g)	18.4 ± 1.9 *	18.7 ± 1.6 *	18.3 ± 1.5 *	21.3 ± 1.6	0.007
MUFAs (g)	22.9 ± 1.7 *	19.5 ± 1.5	19.4 ± 1.6	17.9 ± 1.5	0.008
PUFAs (g)	6.8 ± 0.9	7.2 ± 1.2 *°	6.3 ± 0.6	6.2 ± 0.5	0.004
Total dietary cholesterol (mg)	190.1 ± 14.6 *	199.2 ± 15.3 *	194.3 ± 16.2 *	229.4 ± 17.2	0.003
Total dietaryfiber (g)	20.9 ± 2.3 *	18.2 ± 1.4	17.9 ± 1.5	16.9 ± 1.9	0.009

Values are expressed as the median (interquartile range); Analysis of Variance—ANOVA followed by Tukey post-hoc test: * *p* < 0.05 vs. animal fats, ° *p* < 0.05 vs. other vegetable oils. SFAs = Saturated fatty acids, MUFAs = Monounsaturated fatty acids, PUFAs = Polyunsaturated Fatty Acids.

**Table 3:** Haemodynamic characteristics of subjects consuming different predominant cooking fats in every day meals.

	Extra-Virgin Olive Oil (*N*. 501)	Corn Oil (*N*. 253)	Other Vegetable Oils (*N*. 107)	Animal Fats (*N*. 103)	ANOVA *p*-Value
SBP (mmHg)	136.8 ± 10.2 *°	138.4 ± 10.9 *	140.3 ± 11.4	141.6 ± 10.3	0.039
DBP (mmHg)	72.0 ± 3.8	72.5 ± 5.4	73.4 ± 5.0	73.8 ± 4.9	0.776
PP (mmHg)	64.8 ± 4.1 *	65.8 ± 8.5 *	66.9 ± 8.8	67.8 ± 8.3	0.029
MAP (mmHg)	93.6 ± 3.8 *	94.8 ± 6.2	95.7 ± 6.1	96.4 ± 5.9	0.148
ABP (mmHg)	134.3 ± 10.6 *°	136.2 ± 10.7 *	137.0 ± 10.6	138.6 ± 10.3	0.037
APP (mmHg)	62.3 ± 4.8 *	63.5 ± 7.1	63.7 ± 8.6	64.8 ± 8.7	0.147
AIx	24.5 ± 4.6 *°	24.9 ± 4.7 *	26.2 ± 4.3 *	30.0 ± 3.8	0.009
cfPWV (m/s)	8.6 ± 0.6 *	8.7 ± 1.0 *	8.9 ± 1.1	9.3 ± 1.3	0.018
Heart Rate (bpm)	65.1 ± 3.8	63.2 ± 7.4	63.9 ± 6.1	64.1 ± 5.6	0.765

* *p* < 0.05 vs. Animal fats; ° *p* < 0.05 vs. Other vegetable oils (Analysis of Variance—ANOVA followed by Tukey post-hoc test; all variable were normally distributed). SBP = Systolic Blood Pressure, DBP = Diastolic Blood Pressure, PP = Pulse Pressure, MAP= Mean Arterial Pressure, ABP = Aortic Blood Pressure, APP = Aortic Pulse Pressure, Aix = Augmentation Index, cfPWV = carotid-femoral Pulse Wave Velocity.

**Table 4:** Glyco-lipidic characteristics of subjects predominantly consuming different cooking fats in everyday meals.

	Extra-Virgin Olive Oil (*N*. 501)	Corn Oil (*N*. 253)	Other Vegetable Oils (*N*. 107)	Animal Fats (*N*. 103)	ANOVA *p*-Value
FPG (mg/dL)	86.9 ± 6.5 *^§^°	94.5 ± 7.9	96.0 ± 11.3	95.4 ± 9.2	0.027
TC (mg/dL)	216.3 ± 19.9 *	212.4 ± 21.5 *	219.1 ± 19.8 *	235.0 ± 26.1	0.034
LDL-C (mg/dL)	140.9 ± 18.8 *	138.6 ± 19.9 *	143.3 ± 18.3	149.2 ± 17.3	0.039
TG (mg/dL)	99.1 ± 24.9 *	110.9 ± 26.3	118.6 ± 36.8	119.7 ± 34.6	0.088
HDL-C (mg/dL)	66.0 ± 10.5 *^§°^	50.1 ± 7.3	51.4 ± 7.0	51.9 ± 7.9	0.074
ApoB (mg/dL)	91.8 ± 10.3	90.3 ± 10.7 *	92.2 ± 10.1	94.4 ± 11.1	0.119
ApoAI (mg/dL)	163.0 ± 13.4 *^§^°	153.2 ± 14.2	152.1 ± 14.7	154.3 ± 14.1	0.108
Lp(a) (mg/dL)	21.0 (3.1–47.4) *	24.4 (4.3–41.7) *	21.3 (5.7–44.3) *	33.9 (6.1–48.4)	0.003

* *p* < 0.05 vs. Animal fats; ^§^ *p* < 0.05 vs. Corn oil, ° *p* < 0.05 vs. Other vegetable oils (Analysis of Variance—ANOVA followed by Tukey post-hoc test; all variable were normally distributed but Lp(a), reported as median and 95% confidence intervals). FPG = Fasting Plasma Glucose, FPI= Fasting Plasma Insulin, TC = Total Cholesterol, LDL-C = Low Density Lipoprotein Cholesterol, TG = Triglycerides, HDL-C = High Density Lipoprotein Cholesterol, ApoB = Apolipoprotein B100, ApoAI = Apolipoprotein AI, Lp(a) = Lipoprotein(a).

**Table 5:** Liver and renal characteristics of subjects predominantly consuming different cooking fats in everyday meals.

	Extra-Virgin Olive Oil (*N.* 501)	Corn Oil (*N.* 253)	Other Vegetable Oils (*N.* 107)	Animal Fats (*N.* 103)	ANOVA *p*-Value
AST (U/L)	24.8 ± 5.3	22.9 ± 7.2	23.6 ± 10.8	26.0 ± 9.5	0.583
ALT (U/L)	23.5 ± 9.8	25.7 ± 10.8	24.6 ± 12.8	24.3 ± 10.8	0.548
gGT (U/L)	26.4 ± 7.7	27.2 ± 9.3	25.2 ± 10.3	27.7 ± 13.0	0.634
HSI	34.4 (27.2–41.4) *	36.9 (27.7–42.4)	37.1 (28.9–43.2)	38.8 (31.4–45.3)	0.132
SUA (mg/dL)	4.9 ± 0.6 *°	5.2 ± 0.9	5.4 ± 1.1	5.3 ± 1.2	0.091
eGFR (ml/min)	67.5 ± 5.5	71.9 ± 8.3	72.4 ± 7.8	69.9 ± 7.2	0.102

* *p* < 0.05 vs. Animal fats; ° *p* < 0.05 vs. Other vegetable oils (Analysis of Variance—ANOVA followed by Tukey post-hoc test; all variable were normally distributed but HSI, reported as median and 95% confidence intervals). ALT = alanine aminotransferase, AST = aspartate aminotransferase, g-GT = γ-glutamyltransferase, HIS = hepatic steatosis index, SUA = serum uric acid, eGFR = estimated glomerular filtration rate.

## Appendix A

Brisighella Heart Study Group: Marilisa Bove, Arrigo F.G. Cicero, Sergio D’Addato, Elisa Grandi, Federica Fogacci, Marina Giovannini, Elisabetta Rizzoli, Fulvio Ventura, Pierangelo Coppola, Eugenia Ianniello, Mario Soldati, Federica Mariasole Piani, Ilaria Ricci Iamino, Silvia Palmisano, Matteo Landolfo, Martina Rosticci, Giuseppe Derosa, Stefano Bacchelli, Claudio Borghi.
